# Green Synthesis of Ni@PEDOT and Ni@PEDOT/Au (Core@Shell) Inverse Opals for Simultaneous Detection of Ascorbic Acid, Dopamine, and Uric Acid

**DOI:** 10.3390/nano10091722

**Published:** 2020-08-31

**Authors:** Pei-Sung Hung, Guang-Ren Wang, Wei-An Chung, Tze-Ting Chiang, Pu-Wei Wu

**Affiliations:** Department of Materials Science and Engineering, National Chiao Tung University, Hsinchu 300, Taiwan; hps198810@hotmail.com (P.-S.H.); gramwang@gmail.com (G.-R.W.); fsmuniverse42@gmail.com (W.-A.C.); jk25645930702@yahoo.com.tw (T.-T.C.)

**Keywords:** colloidal crystals, inverse opals, Au nanoparticles, PEDOT, electrochemical sensing, ascorbic acid, dopamine, uric acid

## Abstract

We demonstrate a water-based synthetic route to fabricate composite inverse opals for simultaneous detection of ascorbic acid (AA), dopamine (DA), and uric acid (UA). Our process involves the conformal deposition of poly(3,4-ethylenedioxythiophene) (PEDOT) and PEDOT/Au on the skeletons of Ni inverse opals via cyclic voltammetric scans (CV) to initiate the electropolymerization of 3,4-ethylenedioxythiophene (EDOT) monomers. The resulting samples, Ni@PEDOT, and Ni@PEDOT/Au inverse opals, exhibit a three-dimensional ordered macroporous platform with a large surface area and interconnected pore channels, desirable attributes for facile mass transfer and strong reaction for analytes. Structural characterization and material/chemical analysis including scanning electron microscope, X-ray photoelectron spectroscopy, and Raman spectroscopy are carried out. The sensing performances of Ni@PEDOT and Ni@PEDOT/Au inverse opals are explored by conducting CV scans with various concentrations of AA, DA, and UA. By leveraging the structural advantages of inverse opals and the selection of PEDOT/Au composite, the Ni@PEDOT/Au inverse opals reveal improved sensing performances over those of conventional PEDOT-based nanostructured sensors.

## 1. Introduction

Ascorbic acid (AA), dopamine (DA), and uric acid (UA) are three important chemicals present in our central nervous system and serum. The AA, also known as vitamin C, plays critical functions in our body including hormonal activation, anti-oxidation, immunity enhancement, and tissue regeneration [1,2]. The typical AA concentration in human serum is between 50–60 μM, and the deficiency of AA is defined to be lower than 11.4 μM [3]. The DA is a vital neurotransmitter for message transfer in the nervous system and the normal level of DA in serum is between 0.01–1 µM [4]. Insufficient DA concentration is an indicator for patients with depression, drug usage, and Parkinson’s disease [5,6,7]. The UA is a product of metabolic breakdown from purine nucleotides, and its normal concentration in serum is 120–420 μM [8]. It is understood that an abnormal UA level is related to illness such as cardiovascular disease, kidney disease, and gout [9,10,11]. The AA, DA, and UA coexist in human blood serum, and therefore, the development of a fast, accurate, and reproducible analytical method is necessary to detect these analytes simultaneously. 

The sensing of AA, DA, and UA is feasible electrochemically because these chemicals exhibit a two-electron oxidation reaction at sufficient oxidizing potentials. However, the challenge is to distinguish them simultaneously with tailored electrode designs because a typical glassy carbon electrode (GCE) reveals similar oxidizing potentials for these three compounds and hence, their resulting voltammetric responses are overlapped, leading to low resolutions and poor sensitivities [12]. To overcome this problem, a wide variety of composites of carbonaceous materials have been studied [13,14,15,16,17]. In addition, organic composites have been explored. For example, the conducting poly(3,4-ethylenedioxythiophene) (PEDOT) and its composites have demonstrated promising potentials due to their unique molecular structure that enables electrostatic interaction between the anionic AA and positively-charged PEDOT, as well as π–π interaction between the positively-charged DA and PEDOT [18,19,20]. These distinct interactions render the separation of AA and DA signal possible. It is noted that PEDOT has been widely used in electrochemical sensing of many chemicals [21,22].

Recently, Au nanoparticles have been shown as an effective electrochemical sensor because of their impressive chemical stability and biocompatibility, as well as superior electric conductivity for electrocatalytic action [23]. Therefore, the Au-decorated PEDOT hybrid, in which the PEDOT provides the selectivity and the Au nanoparticles offer the sensitivity, has demonstrated enhanced sensing performance toward these analytes [24,25]. So far, most of PEDOT/Au composites have been prepared on planar Au and GCE substrates instead of three-dimensional platforms [24,25,26,27]. We recognize that a three-dimensional macroporous structure is able to provide higher sensitivity and lower detection limit due to its excessive surface area and open porous nature. Among many possible three-dimensional structures, the inverse opals reveal a large surface-to-volume ratio, uniform pores with interconnected channels, and robust mechanical property, attributes that are desirable for electrochemical sensing [28,29]. Therefore, we envision that the fabrication of core@shell inverse opals might be a feasible way to improve sensing performances as earlier examples of core@shell nanostructures have reported impressive results in catalysis, sensing, and electrocatalysis [30,31].

To date, the synthesis of PEDOT is often conducted in organic solvents for desirable electric conductivity and porosity [20,32]. In contrast, there are relatively fewer reports about the formation of PEDOT in an aqueous solution because the solubility of 3,4-ethylenedioxythiophene (EDOT) monomers is limited, and the resulting PEDOT structure becomes rather dense with poor conductivity and sensitivity [33]. However, we recognized that the water-based PEDOT synthesis is environmentally friendly and its drawbacks could be alleviated by leveraging the unique features of inverse opals. In this work, we demonstrated the green synthesis of conformal PEDOT and PEDOT/Au coating on three-dimensional ordered macroporous Ni skeletons (Ni@PEDOT and Ni@PEDOT/Au inverse opals, respectively) in an aqueous solution. The resulting sensing performances of Ni@PEDOT and Ni@PEDOT/Au inverse opals on simultaneous detection of AA, DA, and UA were determined by cyclic voltammetry (CV). Comprehensive materials characterization and electrochemical analysis were conducted and discussed. 

## 2. Materials and Methods

### 2.1. Construction of Colloidal Crystals 

Polystyrene (PS) microspheres with an average diameter of 820 nm were synthesized via an emulsifier-free emulsion polymerization process. First, 1000 mL deionized water was deoxygenated in N_2_ at 65 °C for 12 h in a reactor equipped with a bladed pitched paddle impeller. Next, 160 g styrene (99 wt%, Sigma-Aldrich, St. Louis, MO, USA), 0.005 g sodium 4-styrenesulfonate (90 wt%, Alfa Aesar, Haverhill, MA, USA), and 0.2 g potassium bicarbonate (99 wt%, J.T. Baker, Phillipsburg, NJ, USA) were mixed to form a homogeneous solution. Afterward, 0.4 g potassium persulfate (99 wt%, Sigma-Aldrich, St. Louis, MO, USA) was added as the initiator and the solution was stirred at 370 rpm for 16 h at 60 °C. Lastly, the mixture was dried at 65 °C to evaporate residual solvent, and the remaining PS microspheres were re-suspended in anhydrous ethanol at 25 °C and sonicated for 24 h to form a homogeneous PS colloidal suspension of 0.5 wt% loading.

The PS colloidal crystals were constructed by a vertical electrophoresis step in which a 2 × 2 cm^2^ ITO was used as the working electrode, and a 6 × 6 cm^2^ stainless steel plate was employed as the counter electrode. The distance between these two electrodes was 1 cm. At 10 °C and an electric field of 6 V cm^−1^, the PS microspheres were driven toward the working electrode to form highly-ordered close-packed colloidal crystals. Afterward, the PS colloidal crystals were placed in an oven at 90 °C in air for 48 h. 

### 2.2. Preparation of Ni Inverse Opals

We adopted the Watt’s electroplating solution to backfill the PS colloidal crystals with Ni. The electroplating entailed 2 × 2 cm^2^ PS colloidal crystals as the working electrode and a 4 × 4 cm^2^ Ni plate as the counter electrode. The aqueous electrolyte contained 0.5 M NiSO_4_·6H_2_O (99.95 wt%, SHOWA, Gyoda, Saitama, Japan), 0.125 M NiCl_2_·6H_2_O (98 wt%, SHOWA, Gyoda, Saitama, Japan), and 0.3 M H_3_BO_3_ (99.95 wt%, SHOWA, Gyoda, Saitama, Japan). The Ni plating was carried out by imposing a galvanostatic current of 2.5 mA cm^−2^ for 1 h at 25 °C. Afterward, the sample was immersed in toluene (99.5%, J.T. Baker, Phillipsburg, NJ, USA) at 60°C for 48 h, followed by a heat treatment at 350 °C for 2.5 h in 95% Ar + 5% H_2_ to remove the PS colloidal crystals completely to obtain Ni inverse opals. 

### 2.3. Fabrication of Ni@PEDOT and Ni@PEDOT/Au Inverse Opals 

A conformal PEDOT coating was deposited on the Ni inverse opals by carrying out CV scans in a potential range between −0.6 and 1.1 V with a scan rate of 20 mV s^−1^ at 25 °C for 3 cycles. The electropolymerization of 3,4-ethylenedioxythiophene (EDOT) to PEDOT was conducted using 2 × 2 cm^2^ Ni inverse opals as the working electrode, a 2 × 2 cm^2^ Pt plate as the counter electrode, and an Ag/AgCl as the reference electrode. The electrolyte was aqueous solution of 0.05 M EDOT monomer (98 wt%, XR-ITC), 0.1 M lithium perchlorate (95 wt%, Alfa Aesar, Haverhill, MA, USA), and 0.01 M sodium dodecyl sulfate (98.5 wt%, Sigma-Aldrich, Haverhill, MA, USA). The resulting sample was denoted as Ni@PEDOT inverse opals. 

A conformal PEDOT/Au coating was deposited on the Ni inverse opals by conducting CV scans in a potential range between −0.6 and 1.1 V with a scan rate of 20 mV s^−1^ at 25 °C for 3 cycles. The deposition was performed using 2 × 2 cm^2^ Ni inverse opals as the working electrode, a 2 × 2 cm^2^ Pt plate as the counter electrode, and an Ag/AgCl as the reference electrode. The aqueous solution contained 0.05 M EDOT monomer, 0.1 M lithium perchlorate, 0.01 M sodium dodecyl sulfate, and 0.5 mM hydrogen tetrachloroaurate(III) trihydrate (99.99 wt%, Alfa Aesar, Haverhill, MA, USA). The deposition process involved the oxidation of EDOT to PEDOT and the reduction of Au ions simultaneously to form Ni@PDOT/Au inverse opals. For comparison purposes, we also prepared planar Ni@PEDOT films on ITO substrates using identical processes for sequential plating of Ni and PEDOT.

### 2.4. Materials Characterization

A scanning electron microscope (SEM; Hitachi SU-8010, Chiyoda City, Tokyo, Japan) was utilized to observe the morphology of PS colloidal crystals, Ni inverse opals, Ni@PEDOT inverse opals, Ni@PEDOT/Au inverse opals, and planar Ni@PEDOT films. Their atomic ratios were obtained by an energy dispersive X-ray spectroscope (EDS; HORIBA X-max, Kyoto, Japan) equipped on the SEM. In each analysis, five positions were randomly selected for average. Raman spectroscopy analysis was performed using a Jobin Yvon Labram HR 800 microscope (HORIBA Kyoto, Japan) and the excitation source was a 632.8 nm HeNe laser. X-ray photoelectron electron spectroscope (XPS; Thermo Fisher Scientific, ESCALAB Xi^+,^ Waltham, MA, USA) was used to analyze the compositions and oxidation states of constituents in our samples. A surface cleaning step was adopted by ion beam etching before XPS analysis. The complex impedance measurements were carried out via a potentiostat (Princeton Applied Research VersaSTAT4, Oak Ridge, TN, USA) in 0.1 M phosphate-buffered saline (PBS) at the open circuit potential. The amplitude for the electric stimulation was 10 mV, and the frequency range was from 1 to 100 kHz. A 2 × 2 cm^2^ Pt foil was used as the counter electrode. The distance between the working and counter electrode was 1.5 cm. 

### 2.5. Detection of AA, DA, and UA

The sensing behaviors of Ni@PEDOT inverse opals, Ni@PEDOT/Au inverse opals, and planar Ni@PEDOT films for AA, DA, and UA were explored by CV scans using a potentiostat (Princeton Applied Research VersaSTAT4, Oak Ridge, TN, USA). A 2 × 2 cm^2^ Pt foil and a Ag/AgCl were used as the counter and reference electrode, respectively. The CV profiles were recorded between −0.2 to 0.4 V with a scan rate of 20 mV s^−1^ at 25 °C. The sensing characteristic of our samples was determined by their respective first CV profile in 0.1 M PBS (pH 7) with various concentrations of AA, DA, and UA. 

## 3. Results

### 3.1. Fabrication of Colloidal Crystals and Their Inverse opals

**Figure 1:**
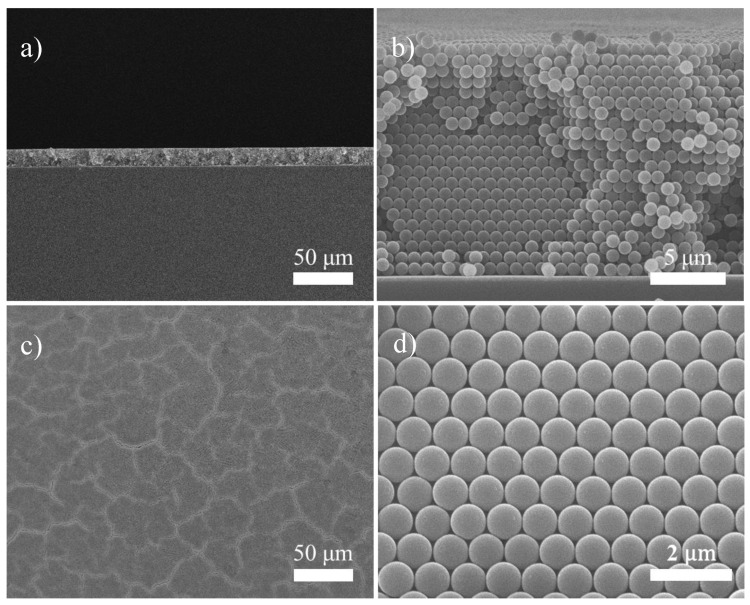
The SEM images for polystyrene (PS) colloidal crystals in cross-sectional view; (**a**) low magnification and (**b**) high magnification, as well as in top view; (**c**) low magnification and (**d**) high magnification.

Colloidal crystals are typically constructed via a self-assembly route, but their small sample size and excess defects render them impractical for engineering applications [34,35]. In our laboratory, we have adopted the electrophoresis to construct colloidal crystals in both planar and cylindrical forms, and relevant electrophoresis parameters have been fully optimized [36,37,38,39]. Moreover, we have fabricated inverse opals in Ni, Au, ZnO, and SiO_2_, and explored their potential applications in chemical sensing and photonic devices [29,40,41,42]. Figure 1 displays the SEM images of PS colloidal crystals in both cross-sectional and top views. As shown in Figure 1a,b the cross-sectional images in low and high magnification confirmed that the PS microspheres were closely packed into a fcc arrangement, and the resulting thickness was around 14 μm, which corresponded to 22 colloidal planes. The SEM top-view image, shown in Figure 1c,d indicated that the colloidal crystals were consisted of many irregularly-shaped grains in size of 40 μm, and the radius of PS microspheres was controlled within a 3% deviation. In addition, the colloidal crystals revealed impressive surface uniformity and negligible crystallographic defect. 

**Figure 2:**
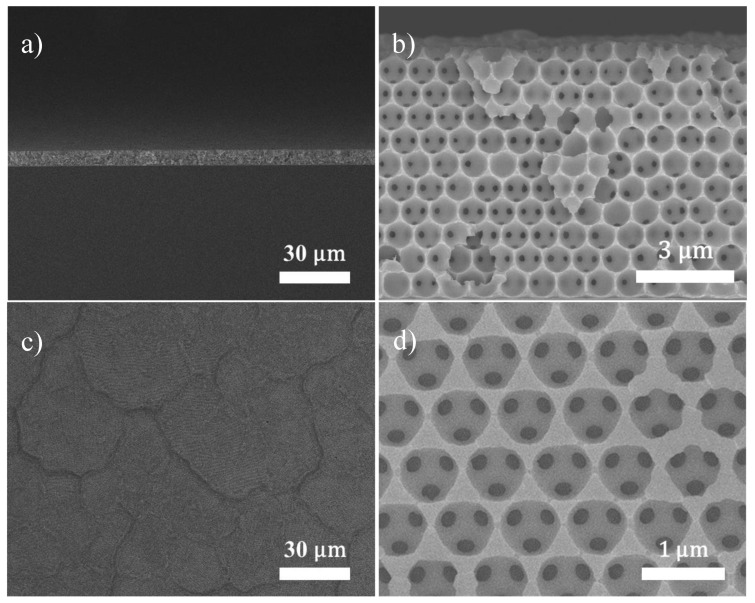
The SEM images for Ni inverse opals in cross-sectional view; (**a**) low magnification and (**b**) high magnification, as well as in top view; (**c**) low magnification and (**d**) high magnification.

The SEM images of Ni inverse opals prepared from filling up the interstitial voids within the PS colloidal crystals are shown in Figure 2. As shown in Figure 2a,b the cross-sectional images in both low and high magnification revealed a three-dimensional ordered macroporous film with a fcc configuration. Notably, these pores were arranged in a honeycomb array in which individual pores were surrounded by twelve neighboring pores with interconnected channels. The presence of these pores was due to the selective removal of PS microspheres in the colloidal crystals. Figure 2c,d display the top-view images in which individual grains in size of 45 μm were observed. This grain size was in reasonable agreement with that of colloidal crystals. In addition, from the enlarged view in Figure 2d, individual pores in the inverse opals were interconnected via smaller pore channels with diameter of 185 nm, a value that was around 1/4 of that of pores. Notably, it was apparent from these images that the Ni inverse opals revealed smooth skeletons with thickness of 80–340 nm. This varying thickness was typical of inverse opaline skeletons [40,41,42]. Lastly, the height for Ni inverse opals was 7.4 μm, which corresponded to 10 inverse opaline layers. With this unique feature of three-dimensional ordered macroporous framework with interconnected pore channels, we would expect the inverse opaline structure to not only provide a large surface area for electrochemical action but also allow for facile mass transport of targeted chemicals.

**Figure 3:**
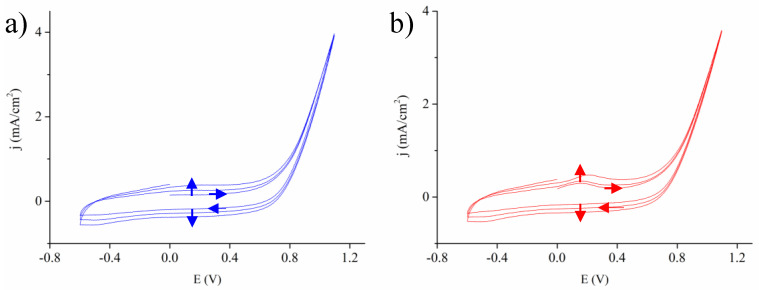
The cyclic voltammetry (CV) profiles for 3 cycles for the conformal deposition of (**a**) PEDOT and (**b**) PEDOT/Au on the skeletons of Ni inverse opals.

In literature, the PEDOT is often synthesized from either a chemical oxidant or an anodic potential in which a radical cation of EDOT is produced, followed by its subsequent reactions with EDOT monomers nearby or neutral oligomeric/polymeric PEDOT for chain propagation [43,44]. Our first attempt entailed a potentiostatic mode at 1.1 V for 2 min which led to a thick PEDOT deposition on the external surface of Ni inverse opals. This excess surface formation of PEDOT failed to leverage the structural advantages of inverse opals. Consequently, we adopted the CV approach with potential scanning from −0.6 to 1.1 V. We rationalized that during the CV scans, the EDOT monomers were allowed to diffuse into the Ni inverse opals in the low voltage regime, followed by electropolymerization on the skeletons in the high voltage regime. Figure 3a displays the CV profiles of PEDOT deposition for three cycles. Apparently, these CV profiles revealed similar patterns in which at the beginning, the current density remained negligible before +0.6 V, and started increasing until it reached to peak at 1.1 V, suggesting the oxidation of EDOT monomers on the Ni inverse opals [33]. In addition, both anodic and cathodic currents became larger with increasing CV cycles, showing a larger PEDOT deposition on the inverse opaline skeletons. It is noted that there appeared redox peaks at 0.2 and −0.4 V, which represented the doping and de-doping of counter anions (ClO_4_^−^) [45,46]. The insertion of counter ions was beneficial for the stabilization of PEDOT in the oxidized state, leading to better conductivity. It is because the PEDOT is known to exhibit a p-type conducting nature upon its oxidation to form polarons and bipolarons with positive charges (holes).

For the deposition of PEDOT/Au, once the Au precursor was mixed with the EDOT solution, the mixture changed its color from transparent to deep blue immediately. This sharp transition indicated the reduction of AuCl_4_^−^ to form Au nanoparticles in conjunction with the oxidation of EDOT to PEDOT oligomer [47]. At this stage, these PEDOT oligomers were able to bond with Au nanoparticles via Au–S interaction so the resulting mixture maintained a homogeneous solution ready for CV cycling [44]. In our observation, the length of PEDOT oligomers was relatively short to allow uninterrupted infiltration into the Ni inverse opals during CV scans. Figure 3b displays the CV profiles of PEDOT/Au deposition for three cycles. Apparently, these CV profiles were rather similar to those of Figure 3a, suggesting that in the electrolyte, despite some preliminary formation of PEDOT oligomers, there remained plenty of residual EDOT monomers to form PEDOT during CV scans. Interestingly, a closer look at 0.2 V showed a slightly larger oxidation current arising from the doping process, indicating a greater doping level of PEDOT/Au. This phenomenon was caused by the rougher and porous PEDOT/Au microstructure as the PEDOT oligomers were impregnated with Au nanoparticles that produced residual pores, engendering a greater surface area for ions doping. 

### 3.2. Materials Characterization of Ni@PEDOT and Ni@PEDOT/Au Inverse Opals

**Figure 4:**
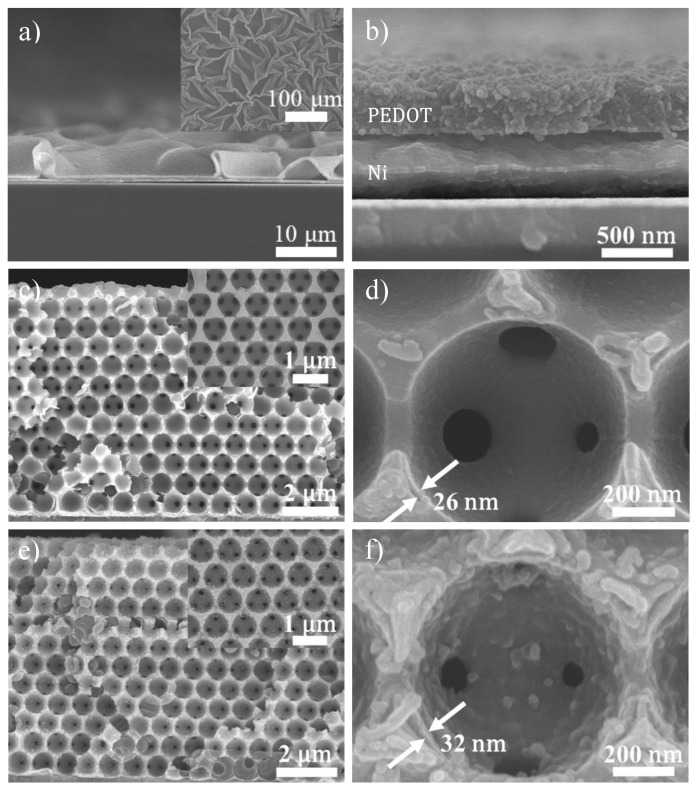
The cross-sectional SEM images for planar Ni@PEDOT films; (**a**) low magnification and (**b**) high magnification, Ni@PEDOT inverse opals; (**c**) low magnification and (**d**) high magnification, and Ni@PEDOT/Au inverse opals; (**e**) low magnification and (**f**) high magnification. The insets are their corresponding top-view images.

Figure 4a,b display the cross-sectional SEM images for planar Ni@PEDOT films in both low and high magnification. As shown in Figure 4a, the sample revealed a rough and wrinkled surface whereas the top-view SEM image (Figure 4a inset) indicated an amorphous and creasy morphology, a pattern that was consistent with what was reported earlier [48]. The enlarged view in Figure 4b confirmed the thickness for the Ni and PEDOT was 400 and 500 nm, respectively. Figure 4c,d display the cross-sectional SEM images for Ni@PEDOT inverse opals in both low and high magnification. Apparently, the Ni@PEDOT inverse opals revealed a honeycomb structure which was identical to that of pristine Ni inverse opals. A closer look in Figure 4d indicated a rough surface, and the resulting thickness for conformal PEDOT coating was around 25 nm. In addition, the diameter for the interconnected pore channels was reduced moderately to 140 nm. From EDS analysis, the atomic content of sulfur, which was indicative of the amount of PEDOT present was merely 1.26%. This value was reasonably expected considering the thickness of the Ni inverse opaline skeleton was between 80 to 390 nm.

Figure 4e,f display the cross-sectional SEM images for Ni@PEDOT/Au inverse opals in both low and high magnification. As shown in Figure 4e the Ni@PEDOT/Au inverse opals revealed a morphology similar to that of Ni@PEDOT, confirming the conformal deposition of PEDOT/Au. This validated our one-step preparation of PEDOT/Au composite in which the Au nanoparticles were uniformly distributed within the PEDOT structure via the Au–S bonding [27]. Consequently, the Ni@PEDOT/Au inverse opals demonstrated an even rougher surface in comparison with that of Ni@PEDOT inverse opals. From Figure 4f, we were able to determine the thickness for the PEDOT/Au coating to be 32 nm, a value that was slightly larger than that of Ni@PEDOT under identical CV cycles. We attributed the relatively greater amount of PEDOT/Au deposition to the higher polymerization degree of PEDOT, which was resulted from the preliminary formation of PEDOT/Au oligomers in the electrolyte and their relatively lower oxidation potential as compared to those of EDOT monomers. In addition, these pre-adsorbed PEDOT/Au oligomers on the Ni inverse opals provided additional nucleation sites for faster deposition of EDOT monomers from the electrolyte. It is noted that the interconnected pore channels of Ni@PEDOT/Au were still evident, albeit with a reduced diameter of 100 nm. This image confirmed that the external surface of the Ni inverse opals and their interconnected pore channels were not blocked by the PEDOT oligomers during EDOT electropolymerization, validating that the size of PEDOT oligomers was relatively small to infiltrate toward the bottom of inverse opaline skeletons for conformal deposition. 

**Figure 5:**
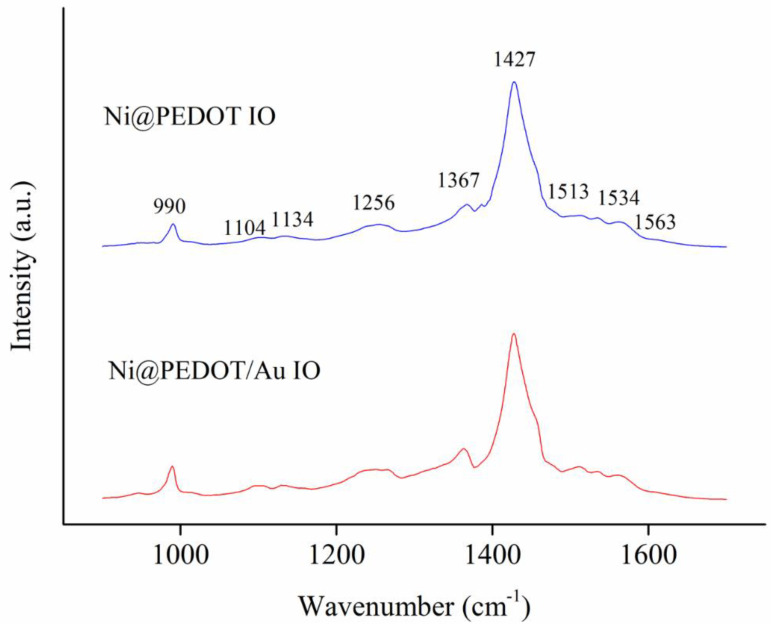
The Raman spectra for Ni@PEDOT and Ni@PEDOT/Au inverse opals, respectively.

To further validate the successful deposition of PEDOT on the Ni inverse opals, we employed the Raman spectroscopy to examine the chemical makeups for both Ni@PEDOT and Ni@PEDOT/Au inverse opals. Figure 5 displays the Raman spectra for both samples. Apparently, they exhibited similar profiles and individual peaks agreed well with what were reported earlier [49,50,51]. Table 1 lists the wavenumbers of Raman signals, and their respective chemical natures. These signals confirmed that our samples were synthesized properly with the expected composition and structure. It is noted that the presence of 1134 cm^−1^ band indicated the formation of NiO [52]. The presence of NiO was attributed to the minor intrinsic surface oxidation of Ni inverse opaline skeletons. 

**Figure 6:**
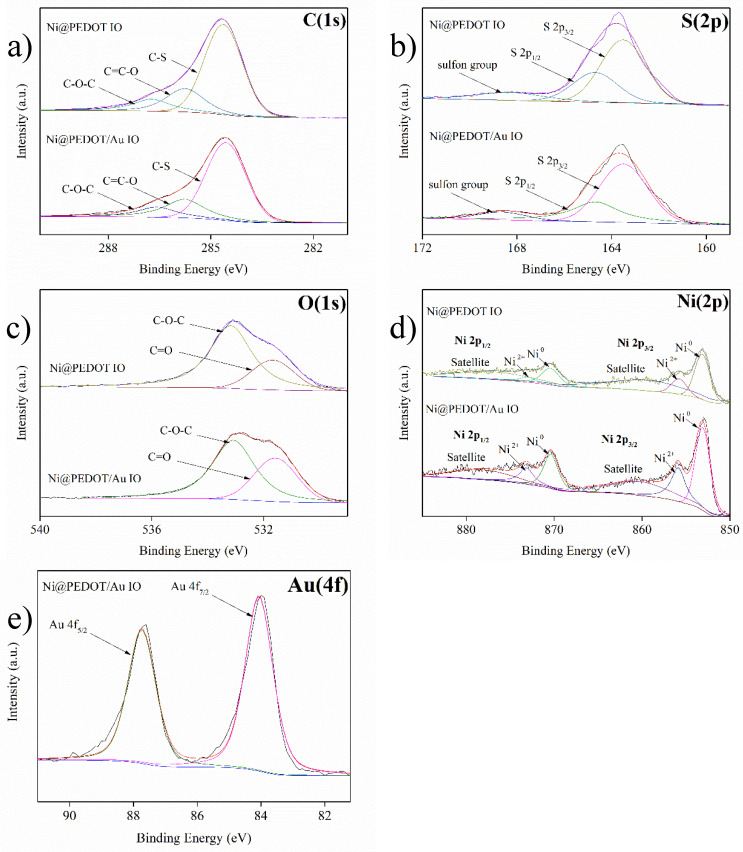
The XPS spectra of (**a**) C(1s), (**b**) O(1s), (**c**) S(2p), (**d**) Ni(2p), and (**e**) Au(4f) for Ni@PEDOT and Ni@PEDOT/Au inverse opals, respectively.

The constituents and their oxidation states for Ni@PEDOT and Ni@PEDOT/Au inverse opals were investigated by XPS analysis and the resulting XPS profiles are exhibited in Figure 6. As expected, the XPS spectra of C(1s), S(2p), O(1s), Figure 6a, the XPS signals were consisted of peaks from three different carbons; 284.6, 285.8, and 286.7 eV, and they were assigned to the C–S, C=C–O, and C–O–C bond in PEDOT [53]. The S(2p) spectra, shown in Figure 6b, demonstrated the characteristic peaks of sulfur in the thiophene ring at 163.5 (S 2p_3/2_) and 164.7 eV (S 2p_1/2_) with a 2:1 intensity ratio [54]. In addition, the minor peak at 168.7 eV was assigned to the sulfon groups from the SDS additive during the electropolymerization of EDOT. For O(1s) spectra shown in Figure 6c, the primary peak at 533.1 eV was associated with the oxygen in dioxy-ring, and the minor one at 531.6 eV was attributed to the oxygen bonding of sulfon groups [54]. For Ni(2p) spectra shown in Figure 6d, the Ni 2p_3/2_ peaks were recorded at 853.1, 855.8, and 859.4 eV, and the Ni 2p_1/2_ peaks were located at 870.4, 873, and 876.7 eV, respectively. The intensity ratio for Ni 2p_3/2_ and Ni 2p_1/2_ was 2:1. From this Ni 2p_3/2_ spectra, the predominant peak at 853.1 eV was assigned to the metallic Ni originating from the Ni inverse opaline skeletons. However, a minor Ni^2+^ signal at 855.8 eV was also recorded, which was caused by the Ni oxidation in ambient atmosphere. The peaks at 859.4 and 876.7 eV were considered to be satellite structures of Ni, a characteristic of surface plasmon loss [55]. Figure 6e displays the spectrum of Au(4f) from the Ni@PEDOT/Au inverse opals. There appeared two noticeable peaks at 84.1 (Au 4f_7/2_) and 87.7 eV (Au 4f_5/2_), and their intensity ratio was 4:3, a value that validated the presence of Au nanoparticles in the composite structure [56]. In short, our results were consistent with what were reported earlier in XPS spectra for both PEDOT and Au, suggesting the successful formation of Ni@PEDOT and Ni@PEDOT/Au inverse opals. 

**Figure 7:**
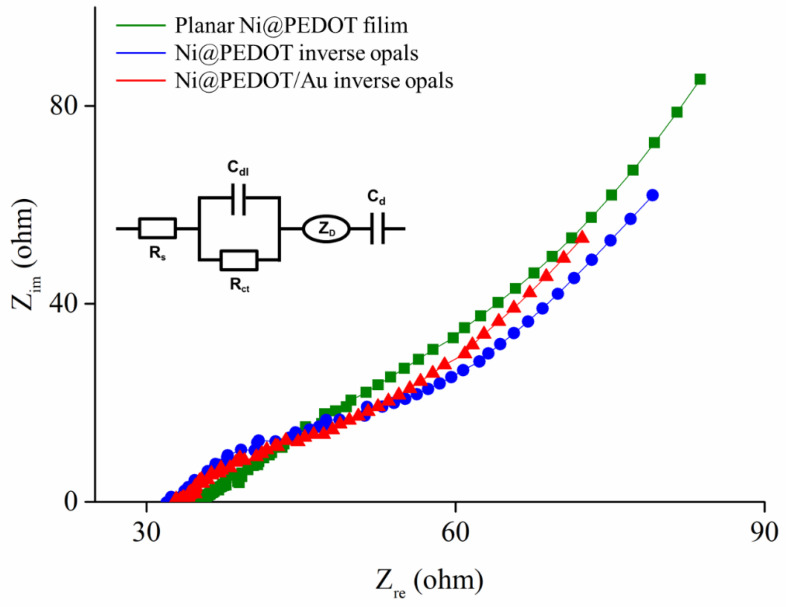
The complex impedance spectra for planar Ni@PEDOT film, as well as Ni@PEDOT and Ni@PEDOT/Au inverse opals in 0.1 M phosphate-buffered saline (PBS).

In electrochemistry, the complex impedance is a powerful analytical tool to extract critical information about the electrode/electrolyte interface. Figure 7 demonstrates the Nyquist plots for Ni@PEDOT and Ni@PEDOT/Au inverse opals, as well as planar Ni@PEDOT film for comparison purpose. The inset illustrates the equivalent circuit model used for the fitting. In impedance spectra, the diameter for the semicircle at the medium-high frequency regime indicates the charge transfer resistance (R_ct_). In addition, the R_s_, the resistance of the electrolyte, is determined by the intercept at the high frequency regime. Other parameters including the double layer capacitance (C_dl_), bulk redox capacitance (C_d_), and the Warburg element (Z_D_) could also be estimated from the equivalent circuit. Table 2 lists the values of R_s_, R_ct_, C_dl_, and R_D_ (diffusion resistance of the Warburg element) by fitting the Nyquist plots with the equivalent circuit model depicted. As listed, the R_s_ value among our samples was around 33 Ω, a value consistent with the conductivity of PBS. In addition, the planar Ni@PEDOT film revealed the lowest R_ct_, the highest C_dl_, and the highest R_D_. We surmised that this was caused by the longer PEDOT chain length, as well as thicker and more continuous PEDOT film for better film quality that led to improved conductivity, higher charge storage, and greater diffusion resistance. For both Ni@PEDOT and Ni@PEDOT/Au inverse opals, the former exhibited a larger R_ct_, a lower C_dl_, and a lower R_D_. These characteristics were attributed to the shorter PEDOT chain length and thinner coating thickness on the Ni inverse opaline skeletons, resulting in a mediocre film quality and conductivity. On the other hand, for Ni@PEDOT/Au inverse opals, the incorporation of Au nanoparticles in the PEDOT structure facilitated the EDOT electropolymerization, resulting in longer PEDOT chain length and better conductivity. We believed that these Au nanoparticles acted as the “electron antennae” to promote better electron transfer across the electrode/electrolyte interface, a phenomenon that was observed earlier [24]. Consequently, the Ni@PEDOT/Au inverse opals demonstrated a subdued R_ct_, indicating a faster electron transport. This led to a slightly increased C_dl_ for higher charge storage and increased R_D_. Despite both Ni@PEDOT and Ni@PEDOT/Au inverse opals showed slightly larger R_ct_ than that of planar Ni@PEDOT film, they provided a greater surface area due to their distinct three-dimensional ordered macroporous structure, a desirable merit for electrochemical sensing. 

### 3.3. Electrochemical Detection of AA, DA, and UA

**Figure 8:**
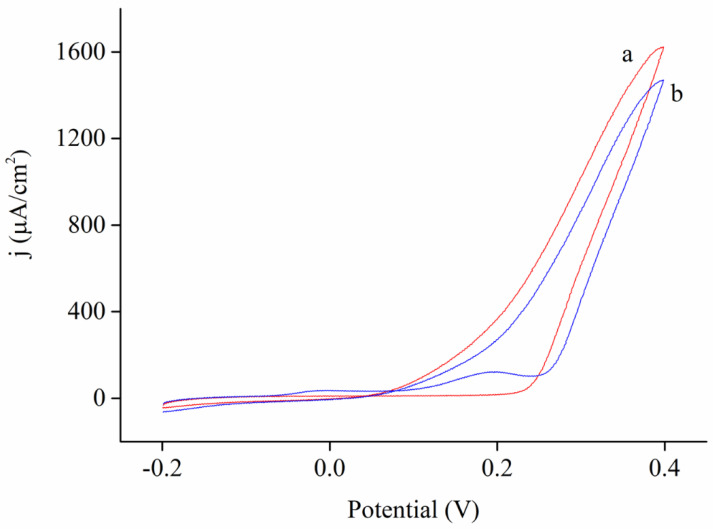
The CV profiles for pristine Ni inverse opals in a) 0.1 M PBS and b) 0.1 M PBS containing 194 μM ascorbic acid (AA), 194 μM dopamine (DA), and 134.4 μM uric acid (UA).

In this work, simultaneous electrochemical detection of AA, DA, and UA were carried out using the CV mode instead of differential pulse voltammetry (DPV) [57]. It is because the DPV mode is not widely available in typical potentiostats so the successful demonstration of CV technique is of practical interest. Because our Ni@PEDOT and Ni@PEDOT/Au inverse opals possess relatively large surface areas as compared to planar counterparts, we recognize that the increased internal surface area could compensate any potential sensitivity loss associated with the CV mode. The electrochemical response of pristine Ni inverse opals was recorded first for any background signal, which was arisen from the Ni inverse skeletons and the ITO substrate underneath. Afterward, the CV scans were conducted in 0.1 M PBS with and without the addition of 194 μM AA, 194 μM DA, and 134.4 μM UA. The resulting CV profiles are displayed in Figure 8. Apparently, from both profiles a notable Ni oxidation was observed at potential larger than 0.2 V. Unfortunately, this Ni oxidation peak overlapped with typical sensing signals associated with AA, DA, and UA. Therefore, we concluded that the pristine Ni inverse opals were unable to detect DD, DA, and UA. Nevertheless, their conductive three-dimensional skeletons with a large surface-to-volume ratio and interconnected pore channels were expected to serve as the ideal scaffold to undergo conformal deposition of PEDOT and PEDOT/Au for enhanced electrochemical responses. 

**Figure 9:**
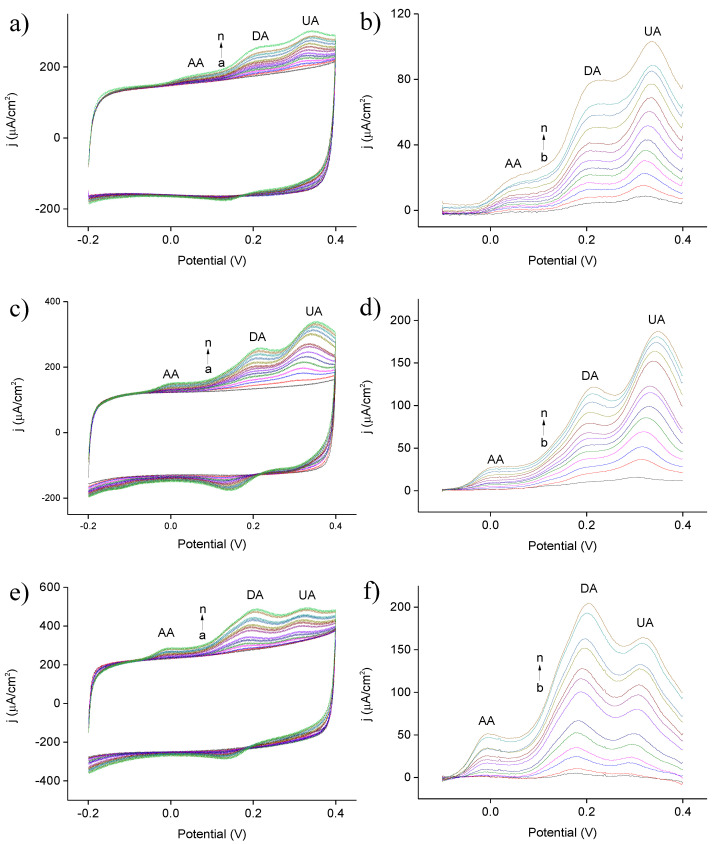
The CV profiles for (**a**) planar Ni@PEDOT film, as well as (**c**) Ni@PEDOT and (**e**) Ni@PEDOT/Au inverse opals, respectively. The electrolyte is 0.1 M PBS containing various concentrations of AA (0–194 μM), DA (0–194 μM), and UA (0–134.4 μM). The exact compositions of individual electrolytes are listed in Table 3. The corresponding background-subtracted CV profiles are displayed in (**b**), (**d**), and (**f**), respectively.

Figure 9a,c,e display the CV profiles for simultaneous detection of AA, DA, and UA using planar Ni@PEDOT film, as well as Ni@PEDOT and Ni@PEDOT/Au inverse opals, respectively. The corresponding concentrations of AA, DA, and UA in individual electrolytes (listed as a→n) are listed in Table 3. Among these CV profiles, there appeared three distinct anodic peaks which were attributed to the oxidation of AA, DA, and UA, respectively. In addition, a notable capacitive current was recorded which was attributed to PEDOT during CV scans. Moreover, a slight shift for the anodic peaks in DA and AA was observed with increasing concentrations of AA, DA, and UA. We realized that the oxidation products of AA were re-adsorbed on the surface of PEDOT, rendering the oxidation of DA and UA to become more anodic [58]. In order to determine the exact oxidation current from these CV profiles, we subtracted the background capacitive current and the resulting background-subtracted CV profiles are exhibited in Figure 9b,d,f The background capacitive current was obtained using the CV profile from 0.1 M PBS without the addition of AA, DA, and UA.

The oxidation potentials for AA, DA, and UA, as well as the corresponding resolution are listed in Table 4. Interestingly, both planar Ni@PEDOT film and Ni@PEDOT inverse opals revealed similar oxidation potentials for DA and UA, and they were located at 0.21 and 0.33 V, respectively. However, for AA, the Ni@PEDOT inverse opals demonstrated a lower oxidation potential at 0.01 V, as compared to that of 0.05 V for planar Ni@PEDOT film. This showed a larger potential difference (resolution) between DA and UA. Clearly, the three-dimensional ordered macroporous structure enabled a better resolution for these three anodic peaks because of a slightly lower oxidation potential of AA. It is noted that the Ni@PEDOT inverse opals demonstrated a better resolution over conventional PEDOT-based electrodes, with oxidation peaks of −0.03, 0.15, and 0.28 V for AA, DA, and UA, respectively. On the other hand, the resolution of Ni@PEDOT/Au inverse opals was slightly worse (0.01 V) than that of Ni@PEDOT inverse opals. However, this minor difference was likely caused by experimental errors as an earlier study from Fabregat et al. determined that the addition of Au nanoparticles in the PEDOT structure had a negligible effect in the resolution [59]. 

**Figure 10:**
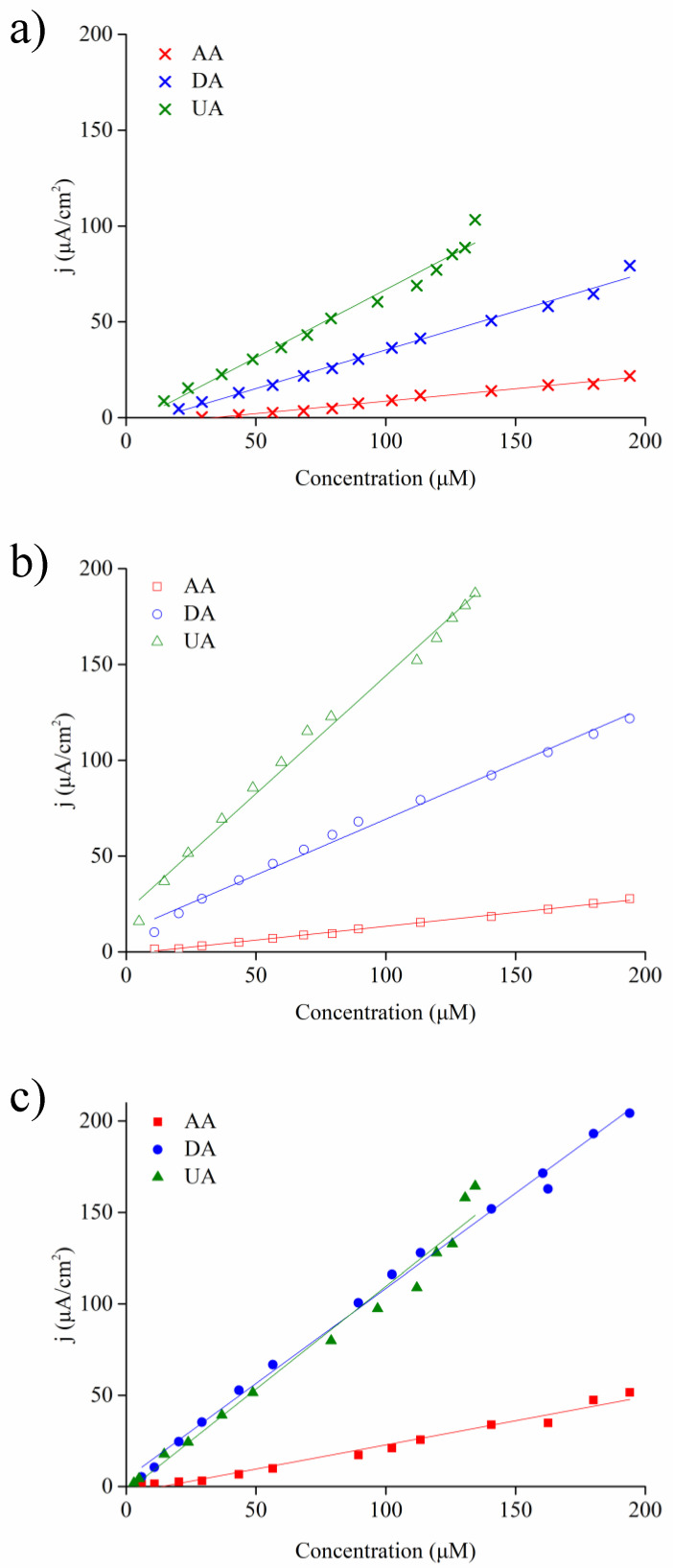
The linear relations of anodic current (after the subtraction of capacitive current) as a function of the concentration of AA, DA, and UA for (**a**) planar Ni@PEDOT film, as well as (**b**) Ni@PEDOT and (**c**) Ni@PEDOT/Au inverse opals, respectively.

Figure 10 displays the linear relations of anodic current (after the subtraction of capacitive current) as a function of the concentration of AA, DA, and UA for planar Ni@PEDOT film, as well as Ni@PEDOT and Ni@PEDOT/Au inverse opals, respectively. These anodic currents were obtained from Figure 9b,d,f and the resulting sensitivities and detection limits are listed in Table 4. The sensitivities were determined by the slope of the fitted linear curve and the detection limit represented the lowest analyte concentration with a noticeable current response during CV scans. For planar Ni@PEDOT film shown in Figure 10a the linear fitted equation for [AA], [DA], and [UA] was j (μA/cm^2^) = 0.13 × [AA] (μM)–4.34 (R^2^ = 0.984), j (μA cm^−2^) = 0.403 × [DA] (μM)–4.95 (R^2^ = 0.99), and j (μA cm^−2^) = 0.709 × [UA] (μM)–4.07 (R^2^ = 0.976), respectively. The corresponding detection limit was 29.22 [AA], 20.3 [DA], and 14.67 μM [UA], respectively. For Ni@PEDOT inverse opals shown in Figure 10b the linear fitted equation for [AA], [DA], and [UA] was j (μA cm^−2^) = 0.145 × [AA] (μM)–1.07 (R^2^ = 0.996), j (μA cm^−2^) = 0.583 × [DA] (μM) + 10.98 (R^2^ = 0.991), and j (μA cm^−2^) = 1.23 × [UA] (μM) + 21 (R^2^ = 0.991), respectively. The corresponding detection limit was 10.88 [AA], 10.88 [DA], and 4.93 [UA] μM, respectively. Despite the planar Ni@PEDOT film revealed a wrinkled morphology for better conductivity, the Ni@PEDOT inverse opals, with a three-dimensional ordered macroporous structure with interconnected pore channels, still outperformed the planar Ni@PEDOT film in both sensitivity and detection limit.

As for Ni@PEDOT/Au inverse opals shown in Figure 10c the linear fitted equation for [AA], [DA], and [UA] was j (μA cm^−2^) = 0.266 × [AA] (μM)–3.65 (R^2^ = 0.978), j (μA cm^−2^) = 1.041 × [DA] (μM) + 4.31 (R^2^ = 0.995), and j (μA cm^−2^) = 1.126 × [UA] (μM)–3.03 (R^2^ = 0.979), respectively. The corresponding detection limits were 5.98 [AA], 5.98 [DA], and 2.98 [UA] μM, respectively. In comparison with those of Ni@PEDOT inverse opals, the Ni@PEDOT/Au inverse opals clearly demonstrated greater sensitivities toward AA and DA, as well as lower detection limits for AA, DA, and UA. This superior performance of Ni@PEDOT/Au inverse opals suggested that the Au nanoparticles in the PEDOT structure facilitated the electron transfer and promoted the oxidation of AA, DA, and UA. 

Table 5 lists the comparison of sensing performances for AA, DA, and UA from planar Ni@PEDOT film, as well as Ni@PEDOT and Ni@PEDOT/Au inverse opals with conventional PEDOT-based sensors reported in the literatures. As listed, with a large surface-to-volume ratio and interconnected pore channels, unique characteristic of three-dimensional ordered macroporous structure, the sensing performances for Ni@PEDOT inverse opals toward the analytes were moderately improved over alternative nanostructured PEDOT-based counterparts. However, after the incorporation of Au nanoparticles, the Ni@PEDOT/Au inverse opals showed enhanced sensitivities toward AA and DA, and better detection limits for AA, DA, and UA. In short, our water-based synthetic route provides an environmentally friendly fabrication process to produce PEDOT-based sensors, and by combining the unique structural features associated with three-dimensional ordered macroporous framework and catalytic activity of Au nanoparticles, we validated the Ni@PEDOT/Au inverse opals to be a promising electrochemical sensor for simultaneous detection of AA, DA, and UA. 

## 4. Conclusions

We demonstrated the conformal deposition of PEDOT and PEDOT/Au on the skeletons of Ni inverse opals using CV cycling in an aqueous solution. The inverse opaline structure allowed for a large surface area with interconnected pores, desirable features for simultaneous detection of AA, DA, and UA. The impregnation of Au nanoparticles in the PEDOT further improved electron transfer, resulting in even better sensing performance as compared to those of conventional nanostructured PEDOT-based counterparts. Our water-based synthesis is environmentally friendly and our results indicated that by combining the structural features of inverse opals and the composite of PEDOT/Au, we were able to overcome the intrinsic limitation of relatively poor PEDOT films from aqueous solution to show better performances over PEDOT-based sensors from more popular organic processing route. In addition, the unique structure of inverse opals made possible the detection of AA, DA, and UA via a straightforward CV technique. 

## Figures and Tables

**Table 1:** The vibration modes of PEDOT in the Raman spectra [46,47,51].

Wavenumbers (cm^−1^)	Assignation
990	Oxyethylene ring deformation
1104	C–O–C deformation
1134	2LO phonon mode of NiO
1256	C_α_-C_α_ (inter-ring) stretching
1367	C_α_–C_β_ stretching
1427	Symmetrical C_α_=C_β_ stretching
1513–1563	Asymmetrical C_α_=C_β_ stretching

**Table 2:** The fitting parameters and their values from impedance spectra of our samples.

	R_s_ (Ω)	R_ct_ (Ω cm^2^)	C_dl_ (10^−3^ F cm^2^)	R_D_ (Ω cm^2^)
Planar Ni@PEDOT film	33.9	5.9	5.4	222.5
Ni@PEDOT inverse opals	32.5	7.6	0.5	94.7
Ni@PEDOT/Au inverse opals	32.7	5.8	0.9	105.1

**Table 3:** The compositions of PBS containing various concentrations of AA, DA, and UA for CV scans displayed in Figure 9. The electrolyte is 0.1 M PBS containing various concentrations of AA (0–194 μM), DA (0–194 μM), and UA (0–134.4 μM).

	Planar Ni@PEDOT Film			Ni@PEDOT Inverse Opals			Ni@PEDOT/Au Inverse Opals		
	AA	DA	UA	AA	DA	UA	AA	DA	UA
a	0	0	0	0	0	0	0	0	0
b	20.30	20.30	14.67	10.88	10.88	4.93	5.98	5.98	2.98
c	29.22	29.22	23.89	20.30	20.30	14.67	10.88	10.88	4.93
d	43.45	43.45	36.90	29.22	29.22	23.89	20.30	20.30	14.67
e	56.50	56.50	48.82	43.45	43.45	36.90	29.22	29.22	23.89
f	68.46	68.46	59.75	56.50	56.50	48.82	43.45	43.45	36.90
g	79.42	79.42	69.77	68.46	68.46	59.75	56.50	56.50	48.82
h	89.47	89.47	78.96	79.42	79.42	69.77	89.47	89.47	78.96
i	102.42	102.42	96.85	89.47	89.47	78.96	102.42	102.42	96.85
j	113.38	113.38	112.00	113.38	113.38	112.00	113.38	113.38	112.00
k	140.70	140.70	119.60	140.70	140.70	119.60	140.70	140.70	119.60
l	162.56	162.56	125.68	162.56	162.56	125.68	162.56	162.56	125.68
m	180.05	180.05	130.55	180.05	180.05	130.55	180.05	180.05	130.55
n	194.04	194.04	134.44	194.04	194.04	134.44	194.04	194.04	134.44

**Table 4:** The oxidation potential and resolution for sensing AA, DA, and UA from planar Ni@PEDOT film, as well as Ni@PEDOT and Ni@PEDOT/Au inverse opals.

Electrode	Oxidation Potential (V)			Resolution (V)			Sensitivity (μA cm^−2^ μM^−1^)			Detection Limit (μM)		
	AA	DA	UA	DA–AA	UA–DA	UA–AA	AA	DA	UA	AA	DA	UA
Planar Ni@PEDOT film	0.05	0.21	0.33	0.16	0.12	0.28	0.13	0.40	0.71	29.22	20.3	14.67
Ni@PEDOT inverse opals	0.01	0.21	0.33	0.20	0.13	0.32	0.15	0.58	1.23	10.88	10.88	4.93
Ni@PEDOT/Au inverse opals	−0.01	0.19	0.30	0.19	0.12	0.31	0.26	1.04	1.13	5.98	5.98	2.98

**Table 5:** The comparison for sensing performances of Ni@PEDOT/Au in this work with other nanostructured PEDOT-based sensors from the literatures.

Electrode	Method	Solvent ^a^	Linear Range (μM)			Sensitivity (μA^2^ μM^−1^ cm^−1^)			[Ref]
			AA	DA	UA	AA	DA	UA	
Ferrocene clicked PEDOT:PSS coated electrode	DC ^b^	H_2_O	N/A	10–900	N/A	N/A	0.196	N/A	[60]
PEDOT-modified Ni/Si MCP electrode	DPV	acetonitrile	20–1400	12–48	36–216	0.539	5.4	2.2	[58]
PEDOT/PNMPy/PEDOT/Au	CV	acetonitrile	N/A	1–100	N/A	0.194	0.182	1.162	[59]
PEDOT-modified GC	DPV	acetonitrile	500–3500	20–80	20-130	0.057	1.365	1.924	[12]
PEDOT-modified GC	DPV	deep eutectic solvent	50–1600	5–180	5–180	0.086	1.46	0.54	[61]
PEDOT-modified GC	DPV	H_2_O	300–1500	100–500	N/A	0.042	0.078	N/A	[25]
Ni@PEDOT/Au inverse opals	CV	H_2_O	6–194	6–194	3–134.4	0.266	1.04	1.13	this work

a: solvent for electropolymerization; b: direct current.
